# Inter- and Intraspecific Variations in the Pectoral Muscles of Common Chimpanzees* (Pan troglodytes)*, Bonobos* (Pan paniscus)*, and Humans* (Homo sapiens)*

**DOI:** 10.1155/2018/9404508

**Published:** 2018-01-21

**Authors:** J. M. Potau, J. Arias-Martorell, G. Bello-Hellegouarch, A. Casado, J. F. Pastor, F. de Paz, R. Diogo

**Affiliations:** ^1^Unit of Human Anatomy and Embryology, University of Barcelona, C/Casanova 143, 08036 Barcelona, Spain; ^2^Animal Postcranial Evolution (APE) Lab, Skeletal Biology Research Centre, School of Anthropology and Conservation, University of Kent, Canterbury CT2 7NR, UK; ^3^Department of Biology, FFCLRP, University of São Paulo, Avenida Bandeirantes 3900, Ribeirão Preto, SP, Brazil; ^4^Department of Anatomy and Radiology, University of Valladolid, C/Ramón y Cajal 7, 47005 Valladolid, Spain; ^5^Department of Anatomy, Howard University College of Medicine, Washington, DC 20059, USA

## Abstract

We have analyzed anatomic variations in the pectoralis major and pectoralis minor muscles of common chimpanzees* (Pan troglodytes)* and bonobos* (Pan paniscus)* and compared them to anatomic variations in these muscles in humans* (Homo sapiens)*. We have macroscopically dissected these muscles in six adult* Pan troglodytes*, five* Pan paniscus* of ages ranging from fetus to adult, and five adult* Homo sapiens*. Although* Pan troglodytes* are thought to lack a separate pectoralis abdominis muscle, we have identified this muscle in three of the* Pan troglodytes*; none of the* Pan paniscus*, however, had this muscle. We have also found deep supernumerary fascicles in the pectoralis major of two* Pan troglodytes* and all five* Pan paniscus*. In all six* Pan troglodytes*, the pectoralis minor was inserted at the supraspinatus tendon, while, in* Pan paniscus* and* Homo sapiens*, it was inserted at the coracoid process of the scapula. Some of the anatomic features and variations of these muscles in common chimpanzees and bonobos are similar to those found in humans, therefore enhancing our knowledge of primate comparative anatomy and evolution and also shedding light on several clinical issues.

## 1. Introduction

The pectoral muscle mass of present-day tetrapods has evolved from the muscle abductor superficialis of the lobed pectoral fin of the Sarcopterygii [1]. This undivided muscle mass was preserved in the last common ancestor of today's tetrapods but later evolved independently into a superficial and a deep layer in the lineage leading to anurans, in the lineage leading to birds plus crocodilians, and in the lineage leading to mammals [2–4]. This division of the pectoral muscle into the superficial pectoralis major and the deep pectoralis minor can be seen in most mammals [4]. In the nonhuman hominoid primates (gibbons, orangutans, gorillas, common chimpanzees, and bonobos), the pectoralis major and minor are relatively large, due to their functional importance in the different types of arboreal locomotion used by these primates. The cranial pectoralis major is active in the protraction of the upper limb during climbing, while the caudal pectoralis major and the pectoralis minor are active during the retraction of the upper limb in the support phase of vertical climbing [5]. In humans, the clavicular head of the pectoralis major protracts the upper limb and the pectoralis minor downwardly rotates and protracts the scapula [6, 7]. When the fully flexed arm is extended against resistance, the sternocostal head of the pectoralis major acts powerfully until the arm reaches the coronal plane [8].

The classic treatises on human anatomy [9, 10] describe in the pectoralis major a clavicular head, a sternocostal head, and an abdominal portion. The clavicular and sternocostal heads are generally present in all hominoid primates, although the clavicular head is not present in orangutans [11]. In contrast, the abdominal portion is absent in 35% of humans [9] and in some cases it can be ignored as a constant portion of the pectoralis major [7]. Some authors identify in hominoid primates a separate pectoralis abdominis muscle, which runs beside the caudal border of the pectoralis major originating from the rectus sheath and being inserted at the crest of the greater tubercle of the humerus [6]. Raven [12] identified a pectoralis abdominis muscle in gorillas, designated as pectoralis quartus, inserted at the coracoid process of the scapula. Ashton and Oxnard [13] argued that the pectoralis abdominis can be identified as a separate muscle from the pectoralis major only in nonhominoid primates, while in hominoid primates, the pectoralis abdominis was a part of the pectoralis major. Moreover, they did not identify this abdominal part of the pectoralis major in humans. Swindler and Wood [14] described neither a separate pectoralis abdominis nor an abdominal portion of the pectoralis major in humans and in common chimpanzees. They did, however, describe a separate pectoralis abdominis in* Papio cynocephalus* inserted at the humerus, the glenohumeral capsule, or the coracoid process. Separate pectoralis abdominis or pectoralis quartus muscles have also been identified in hylobates [15, 16] and in gorillas [12, 17, 18] but not in orangutans [19], common chimpanzees [20], or bonobos.

In humans, the pectoralis minor originates from the third, fourth, and fifth ribs and is inserted at the coracoid process [9]. In hylobates, it is inserted at the coracoid process or the clavicle [21]. In orangutans, the pectoralis minor is inserted at the coracoid process, the proximal humerus, or the glenohumeral capsule [19]. In gorillas and bonobos, it is inserted at the coracoid process [22, 23], and in common chimpanzees at the proximal humerus, the glenohumeral capsule [20], or, less frequently, the coracoid process [13].

Anatomic variations in the pectoral muscles are relatively frequent in humans [24] and can often be related to the characteristics of these muscles in other hominoid primates, such as the presence of the pectoralis abdominis or the variety of insertion sites of the pectoralis minor. Pectoralis abdominis or quartus has been identified in 2.8% [11] to 11–16% [25, 26] of humans. This muscle is considered a segmented portion of the pectoralis major [27], originating from the costochondral joints of the fifth and sixth ribs and the rectus sheath and inserting at the bicipital groove of the humerus or the fascia of the arm [24, 28], the pectoralis major tendon [29, 30], or the coracoid process [31, 32]. A second variation in the pectoralis major is the presence of deep supernumerary fascicles [9] located between the pectoralis major and the pectoralis minor. These fascicles originate from different ribs and are inserted by fusing with the deep surface of the pectoralis major. These fascicles should not be confused with the pectoralis intermedius muscle, which can be also present as a variation and is located between the pectoralis major and the pectoralis minor and which originates from the third and fourth ribs and is inserted at the coracoid process of the scapula [29] or converges with the pectoralis minor [33].

The main variation of the pectoralis minor in humans is its insertion point, which can be the glenohumeral capsule, the greater tubercle of the humerus, or the coracoacromial ligament [9, 34, 35], after passing over the coracoid process and through the coracoacromial ligament [29]. The pectoralis minor can also be inserted at the supraspinatus tendon [29, 36–38]. Magnetic resonance arthrography has identified the insertion of the pectoralis minor at the glenohumeral capsule without insertion at the coracoid process in 1.5% of humans [39], while ultrasound has identified this phenomenon in 9.6% of humans [40].

In the present study we have dissected and analyzed the morphological characteristics of the pectoralis major and minor in common chimpanzees* (Pan troglodytes)* and bonobos* (Pan paniscus)*. Our primary objective is to gain a better understanding of the anatomy of these two muscles and their variations in the two primate species most closely related phylogenetically to humans. The study of the pectoral muscles is especially important in bonobos, because their anatomy and variations have been poorly studied since Miller's classic monograph on the anatomy of the muscles of* Pan paniscus* based on a single specimen [22]. A detailed analysis of the pectoral muscles and their possible variations in common chimpanzees and bonobos will also provide valuable information on the anatomic variations of these muscles in humans, especially the presence of supernumerary muscles, such as the pectoralis abdominis, and the variations on insertion of the pectoralis minor, which have been related to the etiopathogenesis of some diseases such as the subacromial impingement syndrome [35, 37, 38] and to some surgical complications [32, 33, 41].

## 2. Materials and Methods

In this study, we have macroscopically dissected the pectoral region in six common chimpanzees* (Pan troglodytes)*, five bonobos* (Pan paniscus)*, and, for comparison purposes, five humans* (Homo sapiens)*. All specimens were frozen 24 hours post-mortem and stored at −27°C until they were dissected. They were not treated with any fixation method.

The six common chimpanzees (two adult males and four adult females) came from Spanish zoos and were dissected at the Anatomy Museum of the University of Valladolid (Valladolid, Spain). The five bonobos (one adult male, one adult female, one two-month-old male, one eight-month-old male, and one female fetus) came from the Antwerp Zoo (Antwerp, Belgium) and were dissected at the School of Veterinary Medicine of the University of Antwerp (Antwerp, Belgium) as part of the international project: the Bonobo Morphology Initiative. All the apes died from causes unrelated to this study. The five humans (males ranging in age from 81 to 91 years) were cadavers from the Body Donation Service and Dissection Rooms of the University of Barcelona and were dissected at the Anatomy and Embryology Unit of the School of Medicine of the University of Barcelona (Barcelona, Spain) (Table 1).

We first removed the connective and adipose tissue from the pectoral muscles and eliminated the pectoral fascia. We then recorded the anatomic characteristics of the pectoralis major, including its origin and insertion points and the presence of supernumerary muscles (e.g., pectoralis abdominis, pectoralis intermedius) or deep supernumerary fascicles. We then took digital photographs of the muscle with a Canon EOS-50 and finally removed and weighed the muscle. We then removed the surrounding tissue and the clavipectoral fascia from the pectoralis minor and recorded its anatomic characteristics and its origin and insertion points. We took pictures of the muscle with a Canon EOS-50, paying special attention to its insertion points in each specimen. Finally, we removed and weighed the pectoralis minor. In addition, for two specimens, we collected samples of the insertion of the pectoralis minor at the supraspinatus tendon (subject VUPT09, a female common chimpanzee) and at the glenohumeral capsule (subject HS05, a human male). These samples were fixed in a solution of 5% formaldehyde for one week and were prepared for later histological analysis at the Anatomy and Embryology Unit of the School of Medicine of the University of Barcelona with hematoxylin and eosin staining.

## 3. Results

Table 2 displays the main results of the study.

### 3.1. Pectoralis Major

**Figure 1:**
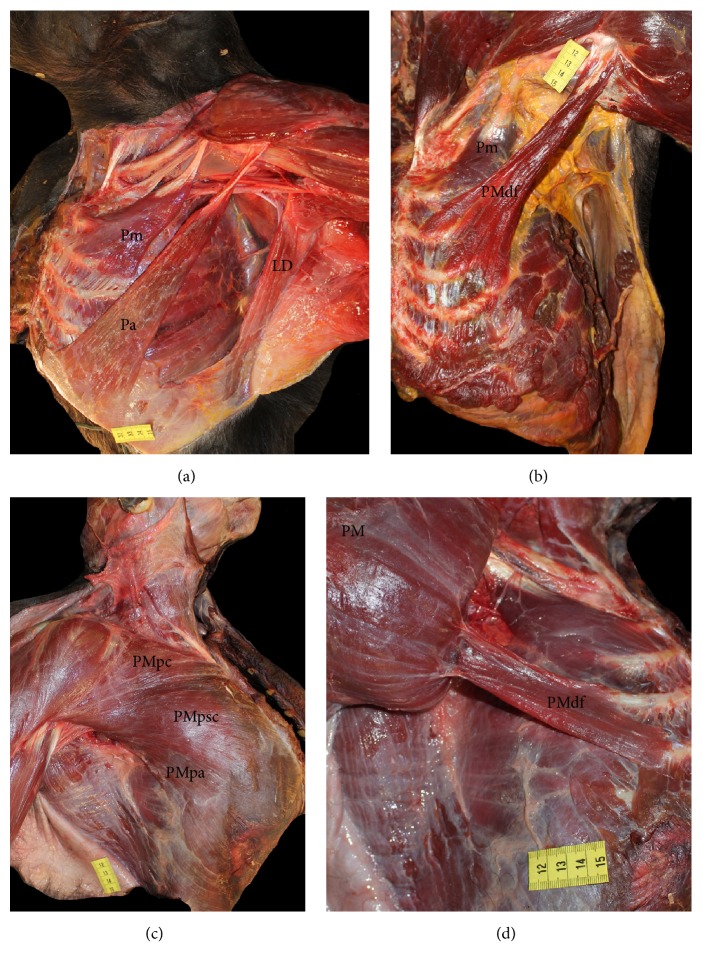
(a) Dissection of a* Pan troglodytes* showing a pectoralis abdominis muscle. Pm (pectoralis minor), Pa (pectoralis abdominis), and LD (latissimus dorsi). (b) Dissection of a* Pan troglodytes*. We can see a deep supernumerary fascicle. Pm (pectoralis minor) and PMdf (pectoralis major deep fascicle). (c) Superficial dissection of the pectoralis major of a* Pan paniscus*. PMpc (pectoralis major pars clavicularis), PMpsc (pectoralis major pars sternocostalis), and PMpa (pectoralis major pars abdominis). (d) Dissection of a* Pan paniscus*. The pectoralis major has been moved to one side to show a deep supernumerary fascicle. PM (pectoralis major) and PMdf (pectoralis major deep fascicle).

In four of the* Pan troglodytes* specimens (VUPT04, VUPT07, VUPT08, and VUPT09), the clavicular head of the pectoralis major originated from the medial half of the clavicle, while, in the remaining two specimens (VUPT05, VUPT06), it originated from the medial two-thirds of the clavicle. The sternocostal head originated from the manubrium and body of the sternum, from the first to seventh costal cartilages (VUPT04, VUPT05, and VUPT06) or the first to sixth costal cartilages (VUPT07, VUPT08, and VUPT09), and from the medial rectus sheath. The abdominal portion of the pectoralis major originated from the lateral rectus sheath and the fascia of the external abdominal oblique muscle. In three specimens (VUPT04, VUPT05, and VUPT06), the abdominal portion completely converged with the sternocostal head, while in the other three specimens (VUPT07, VUPT08, and VUPT09), the abdominal portion was separated from the sternocostal head by a thin layer of areolar connective tissue and formed a clearly identifiable pectoralis abdominis (Figures 1 and 5). This pectoralis abdominis originated from the sixth rib, the rectus sheath, and the fascia of the external abdominal oblique muscle.

In all six* Pan troglodytes* specimens, the pectoralis major was inserted at the crest of the greater tubercle of the humerus. In the three specimens with a clearly identified pectoralis abdominis (VUPT07, VUPT08, and VUPT09), this muscle ran beside the caudal border of the pectoralis major to converge with the dorsal fibers of its insertion tendon. In one specimen (VUPT06), we observed a deep supernumerary fascicle of the pectoralis major (Figures 1 and 5) that originated from the third, fourth, and fifth ribs, near the costochondral junction, and was inserted at the dorsal lamina of the insertion tendon of the pectoralis major. In VUPT07, we also observed a deep supernumerary fascicle of the pectoralis major, but in this case, it was combined with the presence of a pectoralis abdominis. This fascicle originated from the fourth and fifth ribs and inserted at the dorsal lamina of the insertion tendon of the pectoralis major.

In the five* Pan paniscus* specimens, the anatomic distribution of the pectoralis major showed little variation. In two specimens (ZIMS164031, ZIMS164052), the clavicular head originated from the medial one-third of the clavicle, while in the remaining three (ZIMS164041, ZIMS164047, and ZIMS164040), it originated from the medial half of the clavicle. The sternocostal head originated from the manubrium and body of the sternum, at the first to the seventh costal cartilages in three specimens (ZIMS164031, ZIMS164041, and ZIMS164052) and the first to the eighth costal cartilages in the remaining two (ZIMS164047, ZIMS164040), and at the medial rectus sheath. The abdominal portion originated from the lateral rectus sheath and the fascia of the external abdominal oblique muscle. None of the* Pan paniscus* specimens had a pectoralis abdominis separate from the pectoralis major (Figure 1). In all five* Pan paniscus* specimens, the pectoralis major was inserted at the crest of the greater tubercle of the humerus. In three specimens (ZIMS164031, ZIMS164041, and ZIMS164052), we identified a deep supernumerary fascicle of the pectoralis major, located caudally to the pectoralis minor and completely covered by the pectoralis major (Figure 1). In ZIMS164031, this fascicle originated from the fourth and fifth ribs near the costochondral junction, while, in ZIMS164041, it originated from the fifth and sixth ribs and, in ZIMS164052, it originated from the fourth to sixth ribs. In all three cases, this fascicle converged with the deep surface of the sternocostal head of pectoralis major. The remaining two specimens (ZIMS164047, ZIMS164040) had a less differentiated deep supernumerary fascicle, originating from the costochondral junction of the fifth to seventh ribs.

**Figure 2:**
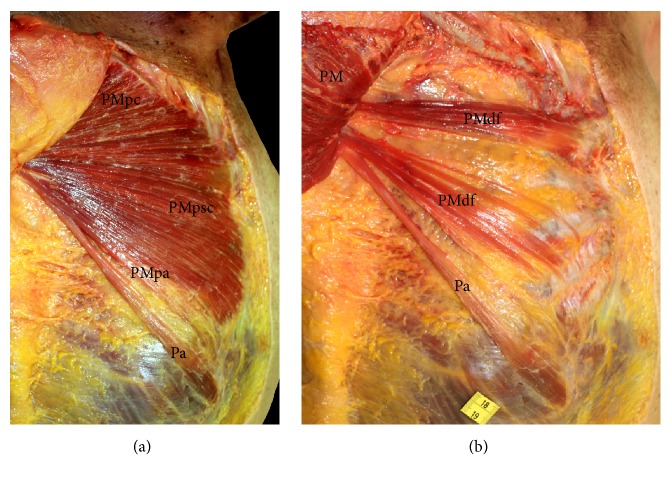
(a) Superficial dissection of the pectoralis major in* Homo sapiens*. We can see a pectoralis abdominis. PMpc (pectoralis major pars clavicularis), PMpsc (pectoralis major pars sternocostalis), PMpa (pectoralis major pars abdominis), and Pa (pectoralis abdominis). (b) Dissection of the pectoral region in* Homo sapiens*. The pectoralis major has been moved to one side to show two deep supernumerary fascicles. PM (pectoralis major), PMdf (pectoralis major deep fascicle), and Pa (pectoralis abdominis).

Among the* Homo sapiens* specimens, the greatest variation in the pectoralis major occurred in three specimens: HS01, HS03, and HS05. In HS01, we observed a Langer's axillary arch extending from the latissimus dorsi to the insertion tendon of the pectoralis major. In HS03, we observed a deep supernumerary fascicle similar to that in the* Pan paniscus* specimen ZIMS164031. This fascicle originated from the costochondral junction of the fourth and fifth ribs and converged with the deep surface of the sternocostal head. In HS05, we observed a highly differentiated pectoralis abdominis (Figure 2) that originated from the rectus sheath and the fascia of the external abdominal oblique muscle and was inserted at the origin tendon of the short head of the biceps brachii. HS05 also had two deep supernumerary fascicles located between the pectoralis major and the pectoralis minor (Figure 2). One of these fascicles, located caudally, was made up of various fascicles that originated from the fourth and fifth ribs and their cartilage. The other fascicle, located cranially, originated from the second rib and its cartilage. Both supernumerary fascicles eventually converged with the deep surface of the pectoralis major.

We also examined other possible anatomic variations in the pectoralis major, including lacking the clavicular head, being inserted at the coracoid process, and converging with the biceps brachii. None of our specimens had any of these variations. In addition, in all the chimpanzees and bonobos in our study, there was a mid-line contact between the two pectoralis major muscles, which was not present in any of the humans.

### 3.2. Pectoralis Minor

**Figure 3:**
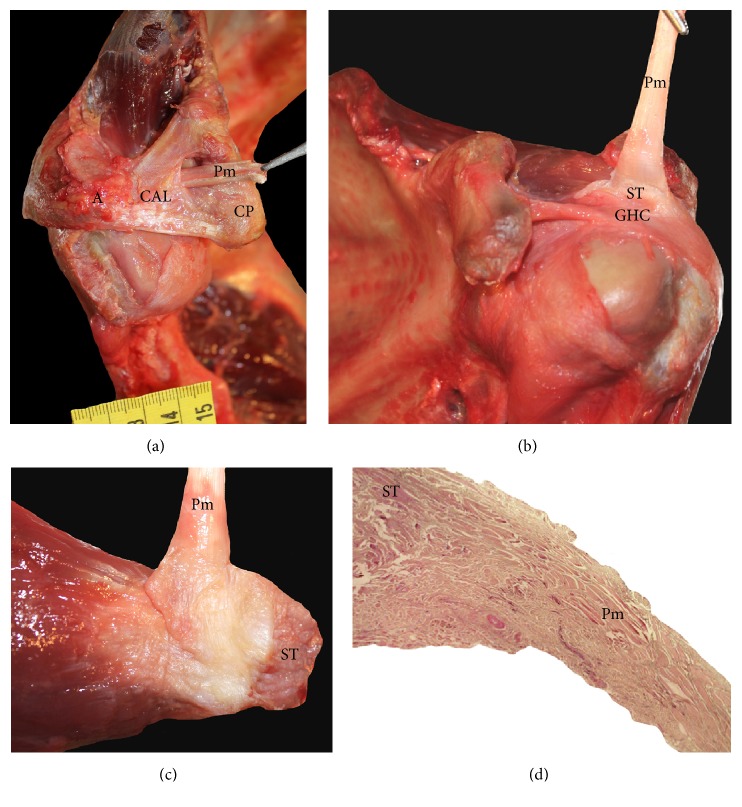
(a) Dissection of a* Pan troglodytes*. We can see the tendon of the pectoralis minor passing through the coracoacromial ligament. Pm (pectoralis minor), CP (coracoid process), CAL (coracoacromial ligament), A (acromion), and S (supraspinatus). (b) Dissection of a* Pan troglodytes*. We can see the insertion of the pectoralis minor tendon in the supraspinatus tendon. Pm (pectoralis minor), ST (supraspinatus tendon), and GHC (glenohumeral capsule). (c) Detailed view of the insertion of the pectoralis minor tendon in the supraspinatus tendon in a* Pan troglodytes*. Pm (pectoralis minor) and ST (supraspinatus tendon). (d) Histological cross section showing the convergence of the pectoralis minor tendon fibers with the supraspinatus tendon in a* Pan troglodytes*. Pm (pectoralis minor) and ST (supraspinatus tendon).

In three* Pan troglodytes* specimens (VUPT04, VUPT05, and VUPT08), the pectoralis minor originated from the second, third, and fourth ribs, while in two specimens (VUPT06, VUPT09), it originated from the second and third ribs, and in the remaining specimen (VUPT07), it originated from the first to fourth ribs. In all six specimens, the insertion tendon of the pectoralis minor passed over the coracoid process and through the coracoacromial ligament between the medial and lateral fascicles (Figure 3), and converged with the supraspinatus tendon (Figures 3 and 5). In four specimens (VUPT05, VUPT06, VUPT07, and VUPT08), we dissected both upper limbs and we found that the pectoralis minor was inserted at the supraspinatus tendon bilaterally. In VUPT09, we confirmed this insertion site by histological analysis (Figure 3). In VUPT07, the insertion tendon crossed the costocoracoid ligament medially to the coracoid process, while in the remaining five specimens, the tendon passed in front of the costocoracoid ligament.

In two* Pan paniscus* specimens (ZIMS164031, ZIMS164052), the pectoralis minor originated from the first to third ribs, while in two specimens (ZIMS164047, ZIMS164040), it originated from the second, third, and fourth ribs and in ZIMS164041, it originated from the first to fourth ribs. In all five specimens, the pectoralis minor was inserted at the coracoid process of the scapula, with a double tendon in two specimens (ZIMS164041, ZIMS164047).

**Figure 4:**
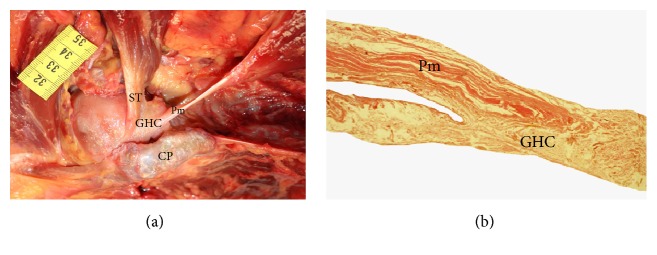
(a) Dissection of a* Homo sapiens*. We can see the insertion of the pectoralis minor in the glenohumeral capsule. Pm (pectoralis minor), CP (coracoid process), GHC (glenohumeral capsule), and ST (supraspinatus tendon). (b) Histological cross section showing the insertion of the pectoralis minor tendon in the glenohumeral capsule in a* Homo sapiens*. Pm (pectoralis minor) and GHC (glenohumeral capsule).

**Figure 5:**
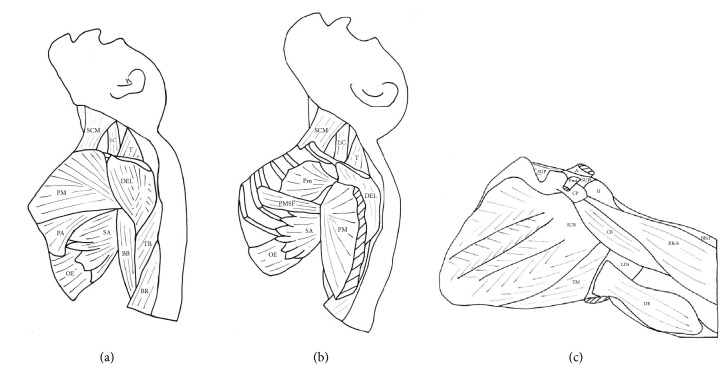
Main muscle variants observed in the present study. (a) Pectoralis abdominis. (b) Deep supernumerary fascicle in the pectoralis major. (c) Insertion of the pectoralis minor at the supraspinatus tendon. SCM (sternocleidomastoideus), LC (levator claviculae), T (trapezius), DEL (deltoideus), PM (pectoralis major), PA (pectoralis abdominis), SA (serratus anterior), OE (obliquus externus), BB (biceps brachii), TB (triceps brachii), BR (brachioradialis), Pm (pectoralis minor), PMSF (pectoralis major supernumerary fascicle), A (acromion), SUP (supraspinatus), SUPt (supraspinatus tendon), Pmt (pectoralis minor tendon), H (humerus), CP (coracoid process), SUB (subscapularis), TM (teres major), LDt (latissimus dorsi tendon), DE (dorsoepitrochlearis), CB (coracobrachialis), BBcb (biceps brachii caput breve), and BBcl (biceps brachii caput longum).

In two* Homo sapiens* specimens (HS01, HS04), the pectoralis minor originated from the second, third, and fourth ribs, while in another two specimens (HS02, HS03), it originated from the third, fourth, and fifth ribs, and in the fifth specimen (HS05), it originated from the second, third, fourth, and fifth ribs. In HS01 and HS05, the insertion tendon split into one medial tendon inserted at the coracoid process of the scapula and a second lateral tendon that passed over the coracoid process and through the lateral and medial fascicles of the coracoacromial ligament and was finally inserted at the glenohumeral capsule (Figure 4). In HS02, a medial tendon of the pectoralis minor was inserted at the coracoid process and a lateral tendon was inserted at the origin point of the coracobrachialis muscle. In HS03 and HS04, the pectoralis minor was inserted at the coracoid process of the scapula.

## 4. Discussion

We have dissected the pectoral muscles of* Pan troglodytes* and* Pan paniscus* and observed a high degree of intra- and interspecies anatomic variation, in line with previous reports on* Homo sapiens* [9, 24, 29]. The frequent variations in these muscles may be related to their complex evolutionary history and embryological development. The pectoral muscles of today's mammals have their origin in a single muscle in the lobed pectoral fin of the Sarcopterygii [1], which later evolved into superficial and deep layers in the evolutionary line that led to the mammals [2–4]. The embryological development of the pectoral muscles takes place in two stages [33]. During the first stage, myogenic cells migrate from the dermomyotome to the precursor of the upper limb, while, in the second stage, these cells return to the trunk, where they anchor the shoulder region to the body wall, forming the pectoral and latissimus dorsi muscles [42–44]. The final anatomic distribution of the pectoral muscles is the result of the three processes of migration, fusion, and apoptosis of the myogenic cells, and any alteration in these processes can modify the origin or insertion points of the muscles or produce supernumerary muscles [33].

In line with previous studies, we have observed variations in the origin of the clavicular and sternocostal heads and the abdominal portion of the pectoralis major muscle in both* Pan troglodytes* and* Pan paniscus*. In* Pan troglodytes*, the clavicular head was reported to originate from the medial fourth, half, or two-thirds of the clavicle [45, 46], while another study reported the absence of a clavicular head [47]. The sternocostal head was reported to extend to the fourth, fifth, sixth, seventh, or eighth costal cartilage [46, 47]. No extensive study has been performed on the anatomical variations of the pectoralis major in* Pan paniscus*, but our findings are similar to Miller's [22] in a single specimen, where the clavicular head originated from the medial half of the clavicle, the sternocostal head extended to the seventh costal cartilage, and the abdominal portion converged with the external abdominal oblique muscle. In our specimens, the clavicular head originated from the medial half or one-third of the clavicle and the sternocostal head extended to the seventh or eighth costal cartilage, while the abdominal portion originated from the fascia of the external abdominal oblique muscle, though the two muscles did not converge.

In the present study, we have found a pectoralis abdominis muscle, separate from the sternocostal head of the pectoralis major, in three* Pan troglodytes* and one* Homo sapiens* but in none of the* Pan paniscus* (Figures 1 and 2). This muscle has not been previously detected in common chimpanzees [20] but has frequently been observed in gorillas [12, 18, 23, 48, 49]. The presence of the pectoralis abdominis in our chimpanzees as an anatomical variation is notable because it can be also present as a variation in* Homo sapiens*, as we observed in HS05. The presence of the pectoralis abdominis as a separate muscle in* Homo sapiens* has clinical implications as it can transpose the medial border of the surgical field (i.e., the caudal border of the pectoralis major) during an axillary lymphadenectomy [28, 41]. In addition, the pectoralis abdominis can compress the axillary neurovascular bundle [32].

A differentiated deep supernumerary fascicle in the pectoralis major (Figure 1) was present in two of our* Pan troglodytes* specimens, one of which also had a pectoralis abdominis muscle. These supernumerary fascicles can be the same muscular variation described previously by MacDowell [45] in a single* Pan troglodytes*. MacDowell [45] stated that in the* Pan troglodytes* specimen dissected by him there is an additional muscle “chondroepitrochlearis” that lies deep into the sternocostal head of the pectoralis major, originates from the second to fourth ribs, and is inserted onto the humerus. The structure that MacDowell designates as “chondroepitrochlearis” is most likely an additional slip of the pectoralis major, and it does not correspond to the variant seen in modern humans referred to as muscle chondroepitrochlearis. In fact, in modern humans this variant muscle usually runs from the ribs to the medial intermuscular septum or onto the medial epicondyle of the humerus [24]. In addition, all of our* Pan paniscus* specimens had a deep supernumerary fascicle that led to a differentiated muscle belly especially in ZIMS164031, ZIMS164041, and ZIMS164052 (Figure 1). The fact that we observed this muscle pattern in all five specimens, ranging in age from fetus to adult, leads us to suggest that this pattern could be a constant anatomic feature of the pectoralis major in* Pan paniscus*. In his classic paper on the muscles of the bonobos, Miller [22] also detected a deep supernumerary fascicle but misidentified it as a pectoralis abdominis.

A differentiated deep supernumerary fascicle in the pectoralis major is a common occurrence in humans. This fascicle originates from one or several ribs and eventually converges with the deep surface of the pectoralis major [9]. One of our* Homo sapiens* specimens (HS03) had a deep supernumerary fascicle with a similar anatomic disposition to that of our common chimpanzees and bonobos. Also, HS05 had one cranial and one caudal deep fascicle (Figure 2), the latter of which had a similar disposition to that of two of our common chimpanzees (VUPT06 and VUPT07) and three of our bonobos (ZIMS164031, ZIMS164041, and ZIMS164052). These deep supernumerary fascicles should not be confused with a pectoralis intermedius muscle, which is also located between the pectoralis major and the pectoralis minor but which is inserted at the coracoid process [29] or the tendon of the short head of the biceps brachii [50] or converges with the pectoralis minor [33]. None of our common chimpanzees, bonobos, or humans had a pectoralis intermedius. It is also important not to confuse the supernumerary fascicles described in the present study with other muscle variants identified in humans, such as the pectoralis minimus or sterno-costo-coracoidian muscle, which extends from the first costal cartilage to the coracoid process [9], or with the chondrofascialis muscle, which extends from the inferior costal cartilages to the medial intermuscular septum [51]. The presence of accessory muscles between the pectoralis major and the pectoralis minor in* Homo sapiens*, whether they are deep supernumerary fascicles or pectoralis intermedius muscles, has clinical implications since they could be mistaken for masses or tumors on computed tomography or magnetic resonance images or could interfere with the surgical use of pectoralis major flaps [33].

The functional importance and clinical interest of the pectoralis major led us to examine other variations in its origin and insertion. For example, the absence of the clavicular head has been reported to be common in orangutans [11] and in some* Pan troglodytes* [47]. In addition, in gorillas, the pectoralis abdominis often is inserted at the coracoid process [12, 48, 49] or converges with the biceps brachii [12, 49]. However, none of our specimens had these characteristics. Furthermore, in all our* Pan troglodytes* and* Pan paniscus*, there was a mid-line contact between the two pectoralis major muscles, which was not present in any of the* Homo sapiens*. This anatomic characteristic is common in most primates but is unusual in humans and in hylobates [18].

The majority of anatomic variations in the origin of the pectoralis minor are related to the number of ribs where the muscle can originate. Previous studies of* Pan troglodytes* found that it originated from the second, third, and fourth ribs [17, 18, 52], the second and third ribs [53–55], the second, third, fourth, and fifth ribs [56], the third, fourth, and fifth ribs [14, 57], or the first, second, third, and fourth ribs [45]. In the present study, we have also observed variations in the origin of the pectoralis minor in* Pan troglodytes*. In three specimens, it originated from the second, third, and fourth ribs but in two specimens, the origin was restricted to only the second and third ribs, and in one specimen, the origin was extended to the first, second, third, and fourth ribs. Few studies have focused on the variations of the origin of the pectoralis minor in* Pan paniscus*, but one report described it as originating from the second, third, and fourth ribs [22]. Our* Pan paniscus* specimens showed a variation similar to that of our* Pan troglodytes*. In two specimens, the pectoralis minor originated from the second, third, and fourth ribs, in two other specimens, the pectoralis minor originated from the first to the third ribs, and in the remaining specimen, the origin was from the first to the fourth ribs. Previous studies in* Homo sapiens* have also detected variations in the origin of the pectoralis minor, ranging from the first to the sixth rib. The most frequently described origin point is the third, fourth, and fifth ribs [9], but we have observed this pattern in only two of our* Homo sapiens* specimens. In two others, the origin was the same as the most common one in our* Pan troglodytes* specimens: the second, third, and fourth ribs. Our fifth* Homo sapiens* specimen had an extended origin: the second, third, fourth, and fifth ribs. Although our sample size is relatively small, our findings suggest that the origin of the pectoralis minor in humans shows a high degree of variation, with examples of an anatomic pattern similar to that of common chimpanzees and bonobos. However, unlike the variations in the pectoralis abdominis and the deep fascicles of the pectoralis major, the variations in the origin of the pectoralis minor in* Homo sapiens* have not been related to clinical implications.

The insertion of the pectoralis minor shows greater variability than its origin in both common chimpanzees and humans. In* Pan troglodytes*, the most commonly observed point of insertion is near the humerus or at the glenohumeral capsule [13, 14, 20], although some studies have reported a complete or partial insertion at the coracoid process of the scapula [46, 48, 56, 58–62]. In the present study, all the* Pan troglodytes* had the same, relatively unusual, pattern of insertion: the tendon of the pectoralis minor passed over the coracoid process and between the lateral and medial fascicles of the coracoacromial ligament (Figure 3) and finally inserted at the supraspinatus tendon, thus attaching indirectly onto the humerus (Figure 3). We observed this pattern in both upper limbs in four* Pan troglodytes* specimens and it was histologically confirmed in VUPT09 (Figure 3). In all our* Pan paniscus* specimens, the pectoralis minor was inserted at the coracoid process of the scapula, which is in line with an early report by Miller [22]. Like the bonobos, the most frequent point of insertion of the pectoralis minor in* Homo sapiens* is the coracoid process of the scapula, though other insertion points have also been reported in 1.5% [39] and 16% [63] of* Homo sapiens*, including the glenohumeral capsule, the greater tubercle of the humerus, and the coracoacromial ligament [9, 29, 35, 39, 64]. In two of our* Homo sapiens* specimens, the pectoralis minor inserted at the glenohumeral capsule (Figure 4) after passing over the coracoid process and through the coracohumeral ligament. The pectoralis minor has also been reported to be inserted at the supraspinatus tendon in humans [29, 36–38, 65], which is similar to our findings in* Pan troglodytes*. Several studies have found that anomalies in the insertion of the pectoralis minor in humans are related to subacromial impingement [66–68], anteromedial subcoracoid impingement [69], and potential compression of the axillary artery and the brachial plexus [35], while others have found no association with clinical symptoms [40]. The insertion of the pectoralis minor in the supraspinatus tendon has been related to shoulder pain and stiffness and to a limited lateral rotation of the glenohumeral joint that can only be corrected by inserting the pectoralis minor in the coracoid process in a surgical intervention [37]. Insertion in the supraspinatus tendon can also hinder the surgical repair of rotator cuff tears [65].

In summary, we have detected a pectoralis abdominis muscle completely differentiated from the pectoralis major in three of our* Pan troglodytes* but in none of our* Pan paniscus* and in only one of our* Homo sapiens* specimens. Differentiated deep supernumerary fascicles of the pectoralis major were present in all our* Pan paniscus* specimens but in only two* Pan troglodytes* and two* Homo sapiens*, leading us to conclude that these fascicles are the norm in* Pan paniscus* but are anatomical variations in* Pan troglodytes* and* Homo sapiens*. Finally, to the best of our knowledge, this is the first study to detect the insertion of the pectoralis minor in the supraspinatus tendon in* Pan troglodytes*, while previous studies have described its insertion at the glenohumeral capsule or the proximal humerus [13, 14, 20]. The pectoralis minor in* Pan paniscus* and* Homo sapiens* generally is inserted at the coracoid process of the scapula but it has also been reported in humans to be inserted at the glenohumeral capsule, the proximal humerus, the coracoacromial ligament, and the supraspinatus tendon. All these anomalies, the pectoralis abdominis [28, 32, 41], the deep fascicles [33], and the insertion points of the pectoralis minor [37, 65–69], have been found to have clinical implications in humans.

## 5. Conclusions

The pectoralis major and pectoralis minor of* Pan troglodytes*,* Pan paniscus*, and* Homo sapiens* have different anatomic patterns with a high degree of variation that may be the result of their evolutionary history and embryological development [44]. The pectoralis major and the pectoralis minor have a species-specific anatomic pattern in common chimpanzees, bonobos, and humans. Although it is generally accepted that the pectoralis abdominis is not present in any of these species, we have detected it in half of the common chimpanzees in the present study. We have observed deep supernumerary fascicles of the pectoralis major in all the bonobos in our study but only as variations in common chimpanzees and humans. The pectoralis minor is inserted at the coracoid process of the scapula in bonobos and humans and at the supraspinatus tendon in our common chimpanzees, which has not previously been described. The pectoralis abdominis, deep supernumerary fascicles, and variations in the insertion of the pectoralis minor have been associated in humans with the development of certain pathologies and with complications in certain surgical procedures. Therefore, the present study not only contributes to the knowledge of primate comparative anatomy and evolution but also sheds light on clinical issues that are important in human medicine.

## Figures and Tables

**Table 1:** Characteristics and sources of specimens included in the study.

Subject	Reference	Sex	Age	Weight (Kg)	Source
*Pan troglodytes *01	VUPT04	Male	Adult (14 years)	82	Madrid Zoo
*Pan troglodytes *02	VUPT05	Female	Adult (26 years)	81.6	Madrid Zoo
*Pan troglodytes *03	VUPT06	Female	Adult (25 years)	42.4	Madrid Zoo
*Pan troglodytes *04	VUPT07	Male	Adult (43 years)	68	Fuengirola Zoo
*Pan troglodytes *05	VUPT08	Female	Adult (40 years)	26	AAP Primadomus, Alicante
*Pan troglodytes* 06	VUPT09	Female	Adult (28 years)	Unknown	Santillana Zoo
*Pan paniscus *01	ZIMS164031	Male	Adult	35	Antwerp Zoo
*Pan paniscus *02	ZIMS164041	Male	Infant (8 months)	2.7	Antwerp Zoo
*Pan paniscus *03	ZIMS164052	Female	Fetus	0.7	Antwerp Zoo
*Pan paniscus *04	ZIMS164047	Female	Adolescent (8 years)	25.7	Antwerp Zoo
*Pan paniscus *05	ZIMS164040	Male	Infant (2 months)	1.9	Antwerp Zoo
*Homo sapiens *01	HS01	Male	Adult (91 years)	Unknown	University of Barcelona
*Homo sapiens *02	HS02	Male	Adult (85 years)	Unknown	University of Barcelona
*Homo sapiens *03	HS03	Male	Adult (81 years)	Unknown	University of Barcelona
*Homo sapiens *04	HS04	Male	Adult (91 years)	Unknown	University of Barcelona
*Homo sapiens *05	HS05	Male	Adult (87 years)	Unknown	University of Barcelona

AAP, Animal Advocacy and Protection.

**Table 2:** Summary of results of the study.

Reference	PA	SFPM	Pm origin	Pm insertion
VUPT04			Ribs 2-3-4	ST
VUPT05			Ribs 2-3-4	ST
VUPT06		X	Ribs 2-3	ST
VUPT07	X	X	Ribs 1-2-3-4	ST
VUPT08	X		Ribs 2-3-4	ST
VUPT09	X		Ribs 2-3	ST
ZIMS164031		X	Ribs 1-2-3	CP
ZIMS164041		X	Ribs 1-2-3-4	CP
ZIMS164052		X	Ribs 1-2-3	CP
ZIMS164047		X	Ribs 2-3-4	CP
ZIMS164040		X	Ribs 2-3-4	CP
HS01			Ribs 2-3-4	CP/GHC
HS02			Ribs 3-4-5	CP
HS03		X	Ribs 3-4-5	CP
HS04			Ribs 2-3-4	CP
HS05	X	X	Ribs 2-3-4-5	CP/GHC

PA = pectoralis abdominis, SFPM = supernumerary fascicle in the pectoralis major, Pm = pectoralis minor, ST = supraspinatus tendon, CP = coracoid process, and GHC = glenohumeral capsule.
